# Rochester Pathological Society

**Published:** 1885-12

**Authors:** Edward Angell


### Rochester Pathological Society.

*Meeting of November 1, 1885.*

Reported by Edward Angell, M. D.

A most instructive paper on epiphyseal fractures was presented by Dr. Richard Moore. Particular attention was called to the necessity, in the case of children, of careful consideration of the possibility of such lesion. Very frequently it has been overlooked, the resulting deformity passed by as a simple sprain or regarded as a luxation of the neighboring joint. Nor must it be forgotten that separation of the epiphysis is possible throughout life in the acromion and coracoid process of the scapula. Indeed, according to Hamilton, fracture of the acromion is always epiphyseal.

In the author's experience, this form of fracture occurs most often in the lower end of the radius, contrary to the statement of Bruns, who gives the first place in frequency to epiphyseal fracture of the lower end of the femur. An explanation of this difference of statement was offered through the probability that lower radial epiphyseal separation was often confounded with Colles' fracture. Union, as a rule, takes place rapidly, if there is true opposition, similar to healing by first intention in the soft tissues. Measurement does not show any shortening as a rule, and, therefore, the union is not of a bony nature. In the author's personal experience, the union is generally cartilaginous, rarely by connective tissue.

The instances of epiphyseal fracture of special interest include those of the acromion, of the upper and lower extremities of the humerus, and of the lower end of the femur. In separation of the acromion, all the treatment required is to keep the patient in bed with the arm at right angles with the body, in order to relax the muscles. Separation of the upper epiphysis of the humerus from the shaft is, again and again, mistaken for luxation, and useless violence resorted to, in order to remedy the obstinate deformity. The diagnosis depends upon the muffled crepitus, but more particularly upon the peculiar resulting deformity which, once seen, can never be forgotten. Robert Smith gives a very lucid and beautiful description of its appearance, but fails utterly to grasp its pathology and treatment.

To reduce the fracture, the arm should be carried *forward* and upward to the vertical position. Then, with steady, but gentle, extension, it should be carried to the side and confined with a Swinburne's extension dressing. In epiphyseal fracture at the lower end of the humerus, the crucial test between that and luxation is in the reduction.

In recent cases, after reduction, a quick shove of the elbow backward, when the arm is flexed at a right angle, will reproduce the deformity in fracture. In old cases of luxation, motion beyond right angle flexion is free, while in fracture the same effort in making flexion is required throughout. Treatment consists in fixation at extreme flexion.

In epiphyseal fracture of the lower end of the radius, the distinction between it and Colles' fracture is rather of scientific than practical interest, since a similar plan of treatment obtains in both.

In epiphyseal separation of the lower end of the femur, the diagnosis depends upon displacement of the upper fragment downward and of the condyles upward. Its treatment differs from that of luxation of the neighboring joint, in requiring fixation at extreme rather than moderate flexion. This extreme flexion is in contradistinction to the teaching of some authorities, who advise full extension; a plan of treatment that does not secure most satisfactory results.

The paper was listened to with marked attention, and elicited considerable discussion, to which Dr. E. M. Moore, Sr., contributed richly from his own large experience in this puzzling class of cases.

